# A bioactive hydrogel patch accelerates revascularization in ischemic lesions for tissue repair

**DOI:** 10.1093/burnst/tkaf005

**Published:** 2025-05-02

**Authors:** Zhuo Liu, Kang Wu, Hong Zeng, Wenxin Huang, Xuemeng Wang, Ying Qu, Chuntao Chen, Lei Zhang, Dongpin Sun, Sifeng Chen, Xiao Lin, Ning Sun, Lei Yang, Chen Xu

**Affiliations:** Department of Physiology and Pathophysiology, School of Basic Medical Sciences, State Key Laboratory of Molecular Engineering of Polymers, Department of Macromolecular Science, Eye Institute and Department of Ophthalmology, Eye & ENT Hospital, Fudan University, 138 Xueyuan Road, Shanghai 200032, P.R. China; Department of Physiology and Pathophysiology, School of Basic Medical Sciences, State Key Laboratory of Molecular Engineering of Polymers, Department of Macromolecular Science, Eye Institute and Department of Ophthalmology, Eye & ENT Hospital, Fudan University, 138 Xueyuan Road, Shanghai 200032, P.R. China; Orthopedic Institute, Department of Orthopedics, The First Affiliated Hospital, Soochow University, 178 East Ganjiang Road, Gusu District, Suzhou 215021, P.R. China; Department of Physiology and Pathophysiology, School of Basic Medical Sciences, State Key Laboratory of Molecular Engineering of Polymers, Department of Macromolecular Science, Eye Institute and Department of Ophthalmology, Eye & ENT Hospital, Fudan University, 138 Xueyuan Road, Shanghai 200032, P.R. China; Department of Physiology and Pathophysiology, School of Basic Medical Sciences, State Key Laboratory of Molecular Engineering of Polymers, Department of Macromolecular Science, Eye Institute and Department of Ophthalmology, Eye & ENT Hospital, Fudan University, 138 Xueyuan Road, Shanghai 200032, P.R. China; Department of Physiology and Pathophysiology, School of Basic Medical Sciences, State Key Laboratory of Molecular Engineering of Polymers, Department of Macromolecular Science, Eye Institute and Department of Ophthalmology, Eye & ENT Hospital, Fudan University, 138 Xueyuan Road, Shanghai 200032, P.R. China; Department of Physiology and Pathophysiology, School of Basic Medical Sciences, State Key Laboratory of Molecular Engineering of Polymers, Department of Macromolecular Science, Eye Institute and Department of Ophthalmology, Eye & ENT Hospital, Fudan University, 138 Xueyuan Road, Shanghai 200032, P.R. China; China Chemicobiology and Functional Materials Institute, School of Chemistry and Chemical Engineering, Nanjing University of Science and Technology, 200 Xiao Ling Wei Street, Xuanwu District, Nanjing 210094, P.R. China; China Chemicobiology and Functional Materials Institute, School of Chemistry and Chemical Engineering, Nanjing University of Science and Technology, 200 Xiao Ling Wei Street, Xuanwu District, Nanjing 210094, P.R. China; China Chemicobiology and Functional Materials Institute, School of Chemistry and Chemical Engineering, Nanjing University of Science and Technology, 200 Xiao Ling Wei Street, Xuanwu District, Nanjing 210094, P.R. China; Department of Physiology and Pathophysiology, School of Basic Medical Sciences, State Key Laboratory of Molecular Engineering of Polymers, Department of Macromolecular Science, Eye Institute and Department of Ophthalmology, Eye & ENT Hospital, Fudan University, 138 Xueyuan Road, Shanghai 200032, P.R. China; Orthopedic Institute, Department of Orthopedics, The First Affiliated Hospital, Soochow University, 178 East Ganjiang Road, Gusu District, Suzhou 215021, P.R. China; Department of Physiology and Pathophysiology, School of Basic Medical Sciences, State Key Laboratory of Molecular Engineering of Polymers, Department of Macromolecular Science, Eye Institute and Department of Ophthalmology, Eye & ENT Hospital, Fudan University, 138 Xueyuan Road, Shanghai 200032, P.R. China; Department of Basic Medicine, Wuxi School of Medicine, Jiangnan University, 1800 Lihu Road, Binhu District, Wuxi, Jiangsu 214122, P.R. China; Orthopedic Institute, Department of Orthopedics, The First Affiliated Hospital, Soochow University, 178 East Ganjiang Road, Gusu District, Suzhou 215021, P.R. China; Center for Health Sciences and Engineering (CHSE), Hebei Key Laboratory of Biomaterials and Smart Theranostics, School of Health Sciences and Biomedical Engineering, Hebei University of Technology, 8 Guangrong Road, Hongqiao District, Tianjin 300131, P.R. China; Department of Physiology and Pathophysiology, School of Basic Medical Sciences, State Key Laboratory of Molecular Engineering of Polymers, Department of Macromolecular Science, Eye Institute and Department of Ophthalmology, Eye & ENT Hospital, Fudan University, 138 Xueyuan Road, Shanghai 200032, P.R. China

**Keywords:** Ion therapy, Acute myocardial infarction, Lower limb ischemia, Angiogenesis

## Abstract

**Background:**

Magnesium ions play crucial roles in maintaining cellular functions. Research has shown that Mg^2+^ can promote angiogenesis, indicating its potential for treating cardiovascular ischemic diseases. However, conventional intravenous or oral administration of Mg^2+^ presents several challenges, including the risk of systemic side effects, diminished bioavailability, and a lack of targeted delivery mechanisms. In this study, we designed an Mg^2+^-releasing adhesive tissue patch (MgAP) that enables the dural release of Mg^2+^ ions.

**Methods:**

A novel MgAP was developed on the basis of ionic crosslinking. Fourier transform infrared spectroscopy confirmed the chemical structure, whereas rheological analysis demonstrated stable mechanical properties and adaptability to dynamic loads. Sustained Mg^2+^ release was quantified over 7 days by inductively coupled plasma–mass spectrometry. In a rat acute myocardial infarction model, we performed echocardiography and strain analysis to assess cardiac function and histological staining to evaluate adverse remodeling. We also verified the proangiogenic effect through *in vitro* tube formation and *in vivo* immunofluorescence assays. Furthermore, transcriptomics and Western blotting were performed to explore the underlying mechanism. Additional assessments were also carried out in a rat model of lower limb ischemia.

**Results:**

Compared with intravenous administration of magnesium chloride, MgAP application effectively improved cardiac function and reduced adverse remodeling in the myocardial infarction rat model. The left ventricular ejection fraction increased by 20.3 ± 6.6%, and the cardiac radial strain improved by 27.4 ± 4.1%. The cardiac fibrosis area and cell apoptosis rate decreased by 10.9 ± 1.2% and 32.1 ± 5.5%, respectively. RNA sequencing analysis also highlighted the upregulation of genes related to cardiac electrophysiological properties, structural and functional intercellular connections, and revascularization. The increased gap junction protein expression and restored local blood supply could contribute to the cardiac repair process posttreatment. The proangiogenic effect of MgAP was also observed in the rat limb ischemia model.

**Conclusions:**

The above results revealed the convincing vascular regeneration effect of an ion therapy-based hydrogel, which enabled the local delivery of Mg^2+^ to the targeted ischemic tissue, aiding in cardiac and lower limb repair. This study presents a novel strategy and highlights its potential for use across various ischemic conditions.

Highlights

- Developed an ion therapy-based tissue patch (MgAP) that exhibited durable and stable release of magnesium ions, tissue-matched degradation, and dynamic adhesive property.
- MgAP patch elevated the local magnesium ion level at targeted ischemic injury site, and avoided the drawbacks of traditional intravenous or oral magnesium administration.
- MgAP effectively promoted endothelial cell tube formation and enhances angiogenesis *in vitro* and *in vivo*, demonstrating potent revascularization in ischemic myocardium and lower limb models.

## Background

Ischemic diseases are characterized by an insufficient blood supply that leads to inadequate oxygen and nutrient delivery to organs. Ischemia of the lower limbs can lead to severe pain, difficulty walking, and even tissue necrosis and amputation. In contrast, ischemic injury to the heart, such as via myocardial infarction (MI), could involve significant loss of myocardial cells, impaired cardiac pump function, pathological myocardial remodeling, and eventually heart failure [1]. Current treatments for ischemic diseases, including pharmacotherapy (e.g. antiplatelet agents, statins), interventional surgery (e.g. angioplasty, stenting), revascularization (e.g. coronary artery bypass grafting, endovascular procedures), and rehabilitation training (e.g. supervised exercise programs) [2–4], have significantly advanced in recent years, improving patient outcomes and quality of life. However, treating ischemic diseases still faces multiple challenges, including delayed diagnosis, limited treatment options, high costs of reconstructive surgeries, complex comorbidities in patients, and the irreversibility of advanced disease stages [5, 6]. Ischemic injury significantly reduces quality of life as the disease progresses and imposes a substantial burden on the health system [7, 8]. Therefore, developing more effective, safer, and economical methods is particularly important. Angiogenesis is a critical process for restoring blood flow to ischemic tissues, facilitating the improvement of tissue perfusion and the repair of damaged tissues and reducing pathological consequences [9]. The development of effective angiogenic treatment strategies not only directly addresses challenges such as myocardial and limb ischemia (LI) but also may lead to significant breakthroughs in the treatment of a wide range of ischemic diseases.

Metal ions, such as magnesium (Mg^2+^), calcium (Ca^2+^), and potassium (K^+^), are essential regulators of cellular functions, including proliferation, migration, and differentiation, and play vital roles in physiological processes [10, 11]. Recent studies have emphasized the potential of these bioactive metal ions in tissue repair [12, 13]. These ions offer significant advantages over traditional therapies, such as recombinant proteins, in terms of cost efficiency and ease of clinical implementation [14, 15]. Furthermore, their stability and reduced clinical risk highlight their potential as a viable alternative for treating ischemic diseases [16]. Among these elements, magnesium (Mg), an essential mineral for human physiology, has been extensively studied for its proangiogenic effects. Research indicates that magnesium ions contribute to vessel formation and repair by influencing endothelial cell functions and may affect processes such as arteriosclerosis, inflammation, and thrombosis [17]. Additionally, magnesium regulates intracellular calcium levels and protects endothelial function, thereby controlling the functions of vascular smooth muscle and endothelial cells [18, 19]. A previous study highlighted the potential risks associated with magnesium deficiency in relation to cardiovascular health, emphasizing its crucial role in supporting vascular health and enhancing angiogenesis [20]. These findings suggest the importance of magnesium in maintaining vascular function and promoting angiogenesis, providing a scientific basis for its application in the prevention and treatment of ischemic diseases. Although there is preliminary evidence in clinical settings that intravenous magnesium sulfate improves the prognosis of patients with ischemic diseases, its effects on angiogenesis remain unclear [21–23]. Moreover, the drawbacks of traditional intravenous or oral magnesium administration [24, 25] include significant side effects (e.g. systemic adverse reactions and potential arrhythmias), limited absorption, lack of targeted delivery, and dosage constraints, which underscore the need for further exploration of the therapeutic potential of magnesium.

Localized administration of Mg^2+^ provides an alternative to ion therapy that could avoid the limitations and side effects of intravenous or oral administration. However, the therapeutic effects of local administration of Mg^2+^ ions on ischemic heart and lower limb diseases have not been revealed. With the continuing development of biomaterials, biodegradable hydrogels with highly hydrated polymeric networks can serve as a delivery system for bioactive ions into ischemic tissues [26, 27]. Starch, a type of nature-derived polysaccharide macromolecule, was approved by the US FDA for pharmaceutical products many years ago [28] and has been extensively used to prepare hydrogels with excellent biocompatible, tissue-adhesive, biodegradable, and tunable mechanical properties for tissue regeneration and repair. It has been reported that starch and ions can form crosslinked hydrogels capable of the sustained release of ions [29]. Recently, we reported that starch can be prepared as a hydrogel adhesive with self-adaptive dynamic stiffness in response to the cyclic deformation of tissue [30]. In this study, we developed a starch-based Mg^2+^-releasing adhesive tissue patch (MgAP) with balanced solid and fluid properties for the localized delivery of Mg ions to ischemic tissues and evaluated its therapeutic effects on ischemic diseases.

This study presents an innovative MgAP specifically engineered for localized magnesium ion delivery and optimized in terms of both viscoelastic properties and stability, thereby offering an effective treatment strategy for ischemic diseases. MgAP demonstrates excellent tissue adhesion and sustained Mg^2+^ ion release. Furthermore, it exhibits high biocompatibility at both the cellular and organ levels. Crucially, our results reveal that MgAP not only significantly enhanced cardiac function and reduced adverse remodeling in a rat MI model but also had a significant therapeutic effect on revascularization in myocardial and LI models, indicating its substantial potential for clinical application in the treatment of ischemic conditions.

## Methods

### Hydrogel preparation

For preparation of MgAP, 6.87 g of MgCl_2_·6H_2_O (analytical purity, Sinopharm Chemical Reagent) was dissolved in 40 ml of deionized water to obtain a precursor solution of Mg^2+^ ions. Waxy starch (8 g, amylopectin >90%, Qinhuangdao Lihua Starch, China) was subsequently added to the Mg^2+^ solution and stirred at 60°C until a transparent and viscous gel formed. Finally, the prepared transparent gel was transferred into an incubator (DHT-250, DOHAO TEST, Shanghai, China) and stabilized at 37°C and 50% relative humidity for 3 days to obtain MgAP. MgAP was cast and gelled in different containers to form samples of the desired geometries and sizes for subsequent characterization.

### Characterization of chemical components and microstructures

Infrared spectra were collected using a Nicolet iS20 FTIR spectrometer (Thermo Fisher Scientific, USA) equipped with a single-bounce diamond stage attenuated total reflectance accessory. The spectra were recorded at a resolution of 2 cm^−1^ over a wavenumber range of 4000–500 cm^−1^, with each sample scanned 32 times to ensure optimal signal-to-noise ratio. The microstructure and energy dispersive spectroscopy (EDS) map of freeze-dried samples precoated with an Au-Pa coating (SC7620, Quorum Technologies, USA) were observed using a field-emission scanning electron microscopy (SEM, Carl Zeiss, Germany).

### Rheological testing

The rheological properties of the hydrogel were characterized using a discovery hybrid rheometer-2 (DHR) instrument (AR2000, Waters TA instrument, USA) equipped with a temperature control unit under oscillation and flow mode. A parallel-plate configuration was employed with a 20-mm diameter plate and a 1-mm gap between the upper and lower plates. Oscillation tests were conducted on the hydrogel samples at 37°C and 1% strain from 0.1 to 10 Hz to determine the storage modulus (G′), loss modulus (G″), and loss factor (tan δ = G″/G′).

### Mechanical testing

Mechanical tests were performed on a universal testing machine (HY10000; Shanghai Precision Instrument). MgAP was molded into a strip with an original length of 30 mm, a width of 9 mm, and a thickness of 4 mm. For the cyclic stretching tests, we first stretched the MgAP strips to a prestretch value of 50% at 300 mm min^−1^, unloaded the strips to 30 mm, and then reloaded them to 37.5 mm (maximum strain = 25%) for 200 cycles at a speed of 75 cycles min^−1^.

### Adhesion testing

The adhesiveness of MgAP to porcine myocardium was measured by a modified method based on ASTM standard C907-17 using the universal testing machine. Fresh porcine myocardium was flattened and fixed on both the upper and lower crossheads in the universal testing machine. A hydrogel with a thickness of 1 mm was placed to cover the porcine myocardium, which was fixed on the lower crosshead. The upper crosshead was subsequently lowered until the contact force reached 0.1 N. After the contact force relaxed to zero, the upper crosshead was set to separate the adhesive interface at a speed of 60 mm/min, and the stress–displacement curve was recorded. Interfacial strength was defined as the maximum stress generated during the separation process, and interfacial toughness was defined as the amount of energy required to detach a unit area of hydrogel (1 mm thick) from the porcine myocardium and was calculated by integrating the stress–displacement curve. Three distinct samples were tested for all the groups.

### Mg^2+^ ion release testing

To characterize the release behavior of Mg^2+^ ions in aqueous solution, the hydrogel samples were immersed in deionized water at 37°C. The extract solution was collected and replenished every day for up to 7 days. The concentration of Mg^2+^ ions in the extracts was measured by inductively coupled plasma–mass spectrometry (ICP–MS, Optima 8000/S10, PerkinElmer, USA).

### Live/dead cell staining

Live/dead cell staining was performed via a calcein-AM/PI kit (Dojindo, Cat. No. C542) according to the manufacturer’s instructions. Human umbilical vein endothelial cells (HUVECs) from the control group and experimental groups were cultured in ECM containing 6% fetal bovine serum (FBS) (ScienCell, Cat. No. 1001) for 7 days. At the termination time, 100 μl of calcein-AM/PI staining working solution was directly added to the adherent cells after they were washed three times with phosphate buffered saline (PBS) and incubated at 37°C for 15 min. The live cells, emitting yellow–green fluorescence, and dead cells, emitting red fluorescence, were observed simultaneously at an excitation wavelength of 490 ± 10 nm. Finally, ImageJ software was used for quantification.

### Myocardial infarction modeling and treatment in rats

A rat model of MI was established by the ligation of the left anterior descending (LAD) branch of the coronary artery. Prior to surgery, all the rats were fasted overnight and then anesthetized using 5% isoflurane (RWD, R510-22-10) in a mixture of oxygen (1 L/min) delivered via a face mask. During surgery, the rats were placed on a heated operating table to maintain body temperature at ~37°C. A small incision was made along the left side of the chest to expose the heart, and the pericardium was carefully dissected. The LAD coronary artery was then identified and ligated using a 7–0 surgical suture. Ligation was performed ~2–3 mm below the left auricle, ensuring complete occlusion of the vessel to induce a transmural MI. Successful ligation of the LAD was confirmed by observing the characteristic ST-segment elevation on the electrocardiogram of the rats, which is a well-established indicator of acute myocardial injury.

Male SD rats were randomly divided into 4 groups: the SHAM group (thoracotomy without LAD ligation), MI group (MI caused by LAD ligation), MI + MgIV group (treated with intravenous MgCl_2_ post-LAD ligation), and MI + MgAP group (treated with MgAP post-LAD ligation). In the MI + MgIV group, following successful ligation of the LAD, magnesium chloride (MgCl₂) was administered intravenously. The dose and time point of administration were selected on the basis of previous studies [31, 32], with a standard dose of 155 mg/kg of body weight given immediately postligation for 7 days. The magnesium solution was prepared in saline, and the injection agent was administered through the tail vein. In the MI + MgAP group, an MgAP (1 cm square in size and 0.5 mm thick) was directly applied to the infarcted myocardium. The patch was secured to the infarct area by self-adhesion, ensuring close contact with the myocardial tissue to facilitate sustained magnesium ion release.

The experimental animals used in this study were purchased from Shanghai Bikaikeyi Co., Ltd, and were uniformly managed by the Laboratory Animal Care Facility of Shanghai Medical College, Fudan University. All animal testing procedures in this study were conducted in accordance with the Guidelines for the Care and Use of Laboratory Animals published by the National Institutes of Health (NIH Publications, eighth edition, 2011).

### Analysis of cardiac function by echocardiography

From the first week to the fourth week postoperation, the left ventricle was examined by echocardiography. The rats with MI were anesthetized with 1.5% isoflurane (RWD, R510-22-10). The echocardiographic examinations were performed using a high-resolution ultrasound imaging system (Vevo 3100) equipped with a MX250 transducer (20 MHz). The procedure followed standard echocardiographic protocols for small animal heart assessment. B-mode and M-mode images were obtained to measure the dimensions of the left ventricle during systole and diastole. Measurements of the left ventricular end-diastolic internal diameter (LVIDd), left ventricular end-systolic internal diameter (LVIDs), end-diastolic volume, and end-systolic volume were recorded. The following echocardiographic parameters were measured to assess left ventricular systolic and diastolic function:

The left ventricular ejection fraction (LVEF) was calculated using the following formula:


$$ LVEF=\frac{EDV- ESV}{EDV}\ast 100\% $$

The left ventricular fractional shortening (LVFS) was calculated as follows:


$$ LVFS=\frac{LVIDd- LVIDs}{LVIDd}\ast 100\% $$

### Strain analysis

Cardiac strain was assessed using Doppler echocardiography with Vevo Strain software. This analysis allows for a detailed assessment of myocardial deformation in various directions. The left ventricle was divided into six segments, namely, the basal, mid-cavity, and apical segments, in both the anterior and posterior walls. Strain was evaluated in two key deformation directions: radial strain, which measures the contraction and expansion of the myocardium in the radial direction (from the center to the periphery of the ventricle), and longitudinal strain, which measures the deformation along the length of the left ventricle (from base to apex). The velocity, displacement, strain, and strain rate for each segment were calculated using the software, providing a comprehensive assessment of myocardial function. The maximum opening wall delay (OWD) was also calculated to evaluate the synchronicity of myocardial motion. The OWD is an indicator of how well the myocardium moves in a coordinated fashion across opposing segments of the left ventricle. Any significant delay in opposing wall motion may indicate dysfunctional myocardial contraction, which could be a result of infarction effects.

### Histological staining and analysis

Histopathological studies were performed to evaluate myocardial tissue damage and repair at the infarction site. On Day 28 postoperation, the heart from animals in each group was harvested and immediately fixed in 4% paraformaldehyde for 24 h. After fixation, the tissue was embedded in paraffin and sectioned into at 5 μm. Hematoxylin and eosin (H&E) staining for general tissue morphology was used to observe MI size and tissue architecture. Masson trichrome staining for collagen deposition was performed to highlight the fibrotic area in the infarct zone, distinguishing it from the viable myocardium. The ventricular wall thickness and the fibrotic area (as a percentage of the total infarction area) were quantified using ImagePro Plus software. The ventricular wall thickness was measured in the infarcted and noninfarcted regions to assess the extent of myocardial remodeling. The fibrotic area was calculated by measuring the total area of fibrosis in the left ventricular infarction site and is expressed as a percentage of the total infarct area.

### Immunofluorescence staining

The hearts of the experimental animals were first frozen in optimal cutting temperature compound (SAKURA, 4583) and sectioned at a thickness of 8 μm. Frozen heart sections were fixed with acetone, permeated with 0.5% Triton X-100 at room temperature for 20 min, and then sealed with PBS containing 10% normal goat serum (Bioss, C01-03001) at room temperature for 1 h. The sections were incubated with primary antibodies overnight at 4°C. On the next day, the sections were incubated with secondary antibodies with fluorescent labels in the dark at room temperature for 1 h before microscopy. The intensity of the fluorescence corresponding to the specific markers was analyzed using ImageJ. Details of the antibodies used are provided in Table 1.

**Table 1:** List of antibodies

Antibodies	Source	Brand	Catalog number
cTnT	Mouse	Invitrogen	MA5-12960
α-SMA	Mouse	Abcam	ab7817
CX43	Rabbit	CST	3512S
vWF	Rabbit	Abcam	ab6994
CD31	Rabbit	Abcam	ab222783
Secondary antibody	CL594-Anti-Rabbit	Proteintech	SA00013-4
Secondary antibody	CL594-Anti-Mouse	Proteintech	SA00006-3
Secondary antibody	CL488-Anti-Rabbit	Proteintech	SA00013-2
Secondary antibody	CL488-Anti-Mouse	Proteintech	SA00013-1

### TUNEL staining

Apoptotic cell death in the infarcted heart tissue was assessed via terminal deoxynucleotidyl transferase dUTP nick-end labeling (TUNEL) staining. The paraffin-embedded sections were soaked twice with xylene and dehydrated with gradient ethanol (100%, 95%, 90%, 80%, 70%). The tissues were treated with Proteinase K working solution for 15–30 min, and 3850 μl of TUNEL reaction mixture (Roche, 11684795910) was added to the samples. The sections were covered with cover glass and placed in a dark wet box at 37°C for 1 h. Apoptotic cells were counted under a fluorescence microscope at an excitation wavelength of 450–500 nm and a detection wavelength of 515–565 nm.

### RNA extraction and sequencing

We used TRIzol reagent (Vazyme, R401-01-AA) to extract total RNA from the infarct site of the rats in each group on the 28th day. Libraries were sequenced on an Illumina sequencing platform (Illumina HiSeq™ 4000, San Francisco, CA, USA). The sequencing results were subjected to quality control. The HISAT2, SAMtools, featureCounts, and limma programs were ultimately used for quantification and discrepancy analysis.

### Real-time polymerase chain reaction analysis

TRIzol reagent (Vazyme, R401-01-AA) was used to extract total RNA from the infarction area. The extracted RNA was reverse-transcribed into complementary DNA (cDNA) using qScript cDNA SuperMix (Quanta Biosciences, Cat# 95048-025) according to the manufacturer’s instructions under the following conditions: 25°C for 5 min, 42°C for 30 min, and 85°C for 5 min. Quantitative polymerase chain reaction (qPCR) was performed using Hieff qPCR SYBR Green Master Mix (Vazyme, Q711-02) for gene expression analysis. The thermal cycling conditions were as follows: holding stage at 50°C for 2 min and 95°C for 10 min, followed by 40 cycles of 95°C for 15 s (denaturation) and 60°C for 1 min (annealing and extension). The melting curve stage was performed with a temperature gradient from 65°C to 95°C at a ramp rate of 0.1°C/s. PCR verification was performed based on the melting curve analysis of the product. The expression of target gene messenger RNA (mRNA) was normalized to *Gapdh* and expressed as the fold change compared with the respective control. The list of primers used in this study is provided in Table 2.

**Table 2:** 5′-3’ qPCR primer sequences

Gene	Upstream primer(5′-3′)	Downstream primer(5′-3′)
*Collagen I*	ATCTCCTGGTGCTGATGGAC	ACCAGGGAAGCCTCTTTCTC
*Collagen III*	GATCCTAACCAAGGCTGCAA	ATCTGTCCACCAGTGCTTCC
*Gapdh*	ACAGTCCATGCCATCACTGCC	GCCTGCTTCACCACCTTCTTG

### Lower limb ischemia model

To induce lower LI, male SD rats were anesthetized with 5% isoflurane and placed in the supine position. The right hind limb was depilated with depilatory cream to ensure proper exposure for surgery. An incision was made on the right hind limb to expose the femoral artery, which was then ligated at both the proximal and distal ends via 6–0 silk sutures. The artery was then excised, and the wound was closed with sutures. A Doppler laser scanner was used to assess blood perfusion in the ischemic hind limb on Days 1, 7, and 14 postsurgery. Blood perfusion in the ischemic limb was measured and compared with that in the contralateral (normal) limb for each rat. The perfused area was quantified via a laser Doppler perfusion imaging system.

### Measurement of Mg^2+^ ion concentrations in tissue and blood

Tissue and blood specimens were harvested from the affected cardiac region or extremity on the 1st or 5th day following surgical intervention for myocardial infarction (MI) and limb ischemia (LI) treatment. The tissue samples were dissolved in nitric acid to obtain an extract solution. The concentrations of Mg^2+^ ions in the extract solution and blood samples were measured via ICP–MS. The mass ratio of Mg^2+^ in the tissue was further calculated by the following equation:



$$ Mass\ ratio=\frac{C_{Mg}\times V}{m_{tissue}}\times 1000\%_{\small0} $$

where *C_Mg_* is the Mg^2+^ concentration in the tissue extract solution, *V* is the volume of extracted solution, and *m_tissue_* is the mass of the corresponding tissue.

### Immunohistochemistry

Paraffin sections of heart tissue were dewaxed at room temperature, hydrated, and processed via heat-induced antigen retrieval. Then, 3% hydrogen peroxide was added, and the sections were incubated at room temperature for 10 min to inactivate endogenous enzyme activity. Goat serum sealer was added at room temperature for 10 min. The tissue incubated with primary antibody in a wet box at 4°C overnight. The next day, the tissue was incubated with secondary antibody at room temperature for 10 min. At 3 to 10 min after the dropwise addition of 100 μl of DAB solution, the samples were observed under a microscope. Staining was terminated when appropriate. The tissues were rinsed with tap water and counterstained with hematoxylin for 1 to 2 min. Subsequently, the tissues were dehydrated, cleared with gradient alcohol and xylene, and photographed under a microscope.

### HUVEC tube-formation assay

The preparation of the patch material extract solution was meticulously conducted as follows: Initially, a precise quantity of MgAP was dissolved in ECM (ScienCell, Cat No. 1001) at a concentration of 0.01 g/ml. This mixture was then incubated at 37°C for 24 h to obtain the material extract. The extract was subsequently centrifuged at 1500 RPM for 10 min. The supernatant was further purified by passing through a 0.22-μm bacterial filter to eliminate bacterial contaminants. To this filtrate, 10% FBS (Sigma–Aldrich, 12003C) was added to enrich the medium. Prior to the experiment, cells were subjected to starvation, and the following day postdigestion, they were seeded at a density of 5x10^4^ cells/well in a culture plate precoated with Matrigel (Corning, 354 248). The cultures were then supplemented either with ECM alone or with medium containing the material extract and incubated for 4 to 6 h. Imaging was performed at consistent intervals on the basis of the cellular growth rate, and the formation of vascular networks was quantitatively assessed using ImageJ software.

### Statistical analysis

All data are shown as the means ± SDs. Statistical analysis for comparisons between two groups was performed using Student two-tailed t-test. For comparisons involving more than two groups, statistical significance was assessed by one-way analysis of variance (ANOVA) followed by an LSD test. Differences were considered to be statistically significant when *P* < 0.05 (^*^). Prism9 was used for mapping analysis. A transcriptome analysis diagram was generated via a free online platform for data analysis and visualization (http://www.bioinformatics.com.cn).

## Results

### MgAP elastic patch exhibited suitable material and biological properties for treating ischemic diseases

#### Material properties of MgAP

**Figure 1:**
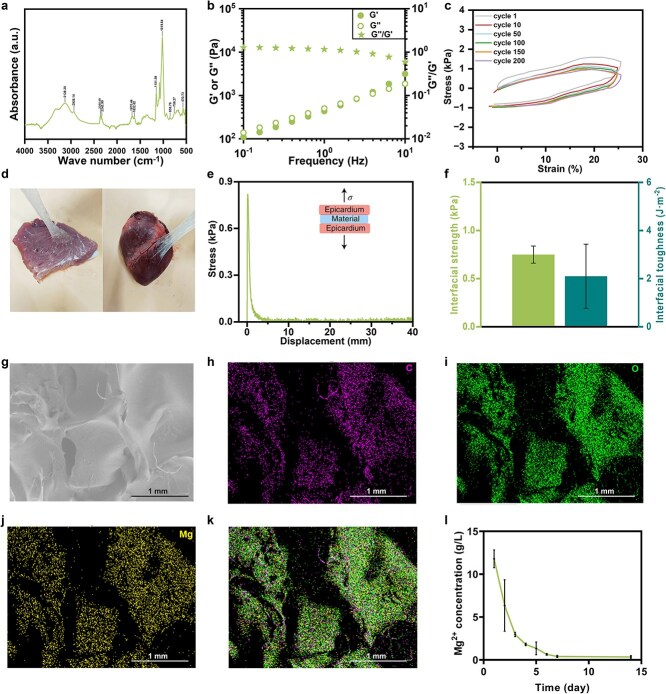
The MgAP elastic patch exhibited appropriate physical properties for treating ischemic diseases. (**a**) FTIR spectrum of MgAP. (**b**) Dependence of G′, G′′, and the G′′/G′ ratio (loss factor) of MgAP on the frequency of oscillation. (**c**) Stress–strain curves of cyclic compressive loading–unloading tests on MgAP. (**d**) Photograph of MgAP adhering firmly to the wet surfaces of skeletal (left) and myocardial (right) muscle. (**e**) Stress–displacement curve from the adhesion test according to a modified method from ASTM standard C907-17, with an inset schematic of the test. (**f**) Strength and toughness of the material–epicardium interface. (**g**) SEM images of freeze-dried MgAP. (**h**–**k**) SEM maps of elemental C, O, and Mg in MgAP. (**l**) Mg^2+^ concentrations in the immersion solution after soaking MgAP for different time periods

To treat ischemic diseases via the localized delivery of Mg ions, a new type of MgAP was developed on the basis of an ionically crosslinked mechanism reported in our previous work [30]. Fourier transform infrared (FTIR) analysis of the chemical structure (Figure 1a) revealed that the characteristic peak of MgAP was assigned mainly to starch, including peaks at 3128.30, 2938.14, 1151.59 and 1019.64 cm^−1^ for the stretching or bending vibrations of –OH, C-H, C-O and C-O-H, respectively, indicating that no chemical reactions occurred between the Mg^2+^ ions and starch. Rheological characterization suggested that MgAP possessed a similar G'' and G' over a wide frequency range from 0.1 to 10 Hz, characterized by a stable constant value of G''/G' near 1 (Figure 1b). The curves of G' and G'' were nearly parallel in a log–log plot of the frequency dependence of the modulus, and both G' and G'' improved with increasing frequency. Importantly, MgAP was able to withstand cyclic loading at the frequency of the dynamic deformation of the human heartbeat (1.25 Hz and strain of 25%), and the stress converged to a steady state within 200 cycles following a rapid decay of stress within the first 10 cycles (Figure 1c). We also demonstrated that the loss, storage modulus and loss factor of MgAP changed only slightly after storage for 60 days (Figure S1). The balanced solid–fluid properties and unique elasticity of MgAP are expected to adapt to the dynamic deformation of soft tissues [30, 33]. In addition to mechanical adaptation, MgAP is also a self-adhesive material that can adhere to the surface of soft tissues (Figure 1d), guaranteeing its suture-free adhesion to dynamically deformed tissues. Taking porcine myocardium as a tissue example, we further quantified its tissue adhesiveness using pull-off adhesion tests (Figure 1e). As shown in Figure 1f, the interfacial adhesion strength and toughness between MgAP and porcine myocardium were 0.75 ± 0.09 kPa and 2.10 ± 1.33 J·m^−2^, respectively. The adhesiveness of MgAP to tissues was attributed to the abundant hydrogen bonds formed between the hydroxyl groups in the amylopectin and the amino groups of the tissue surface [34]. SEM images demonstrated that MgAP possessed a typical porous network structure (Figure 1g). The EDS map (Figure 1h–k) of MgAP revealed a uniform distribution of elemental C, O and Mg within the network. In addition, MgAP was able to release Mg^2+^ ions continuously for 7 days (Figure 1l), which is conducive to vascularization during the early phase of some ischemic diseases, such as MI and critical lower LI [35–37].

#### MgAP exhibited high cell compatibility and enhanced angiogenesis *in vitro*

**Figure 2:**
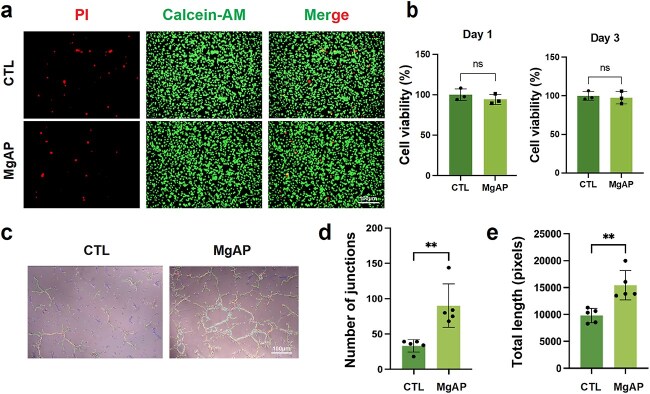
MgAP exhibited satisfactory biosafety and enhanced *in vitro* angiogenesis. (**a**) Live/dead cell staining was conducted after 7 days of the coculture of HUVECs with the MgAP patch extracts. (**b**) CCK-8 assay results showing the viability of cells cultured with the MgAP patch extracts for 1 or 3 days. (**c**) HUVECs were subjected to tube formation assays to assess angiogenic capability. (**d** and **e**) Quantitative analysis of tube formation assay results. (Calcein-AM: Calcein-acetoxymethyl ester, used to stain live cells; PI: Propidium iodide, used to stain dead cells). All data are expressed as the means ± SDs; ns: nonsignificant, *P* > 0.05; ^**^P < 0.01. Statistical analysis for comparisons between two groups was performed using Student two-tailed *t*-test

For tissue repair applications, high biocompatibility is essential. Therefore, we evaluated the biocompatibility of the MgAP patch. HUVECs were cultured with MgAP extracts *in vitro*. Live/dead staining demonstrated the satisfactory cellular compatibility of the patch (Figure 2a). CCK-8 assays performed on Days 1 and 3 of coculture further confirmed that the patch exhibited negligible cytotoxicity **(**Figure 2b). Importantly, the *in vitro* tube formation assays with HUVECs revealed that, compared with the control group, the MgAP group presented significantly faster tube formation and increases in tube number and length (Figure 2c–e). These results underscore the effectiveness of MgAP in promoting endothelial cell alignment and vascular structure formation, suggesting its potential to increase angiogenesis *in vivo*.

### The MgAP elastic patch improved cardiac function in the rat MI model

#### The MgAP elastic patch enhanced left ventricular systolic function following MI injury

**Figure 3:**
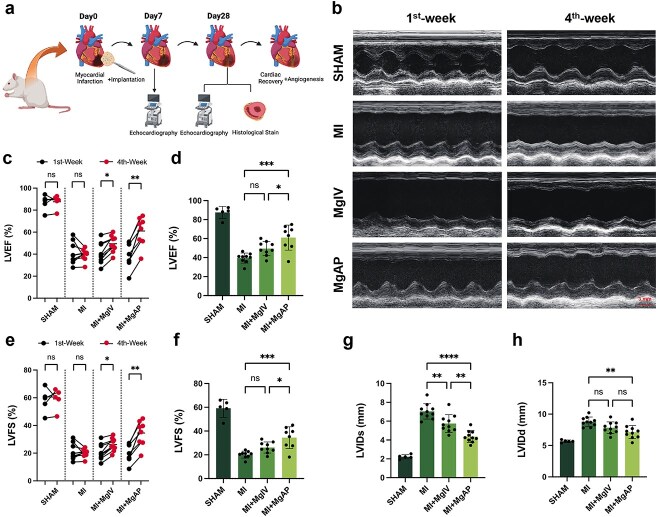
The MgAP elastic patch improved LV systolic function in the rat MI model. (**a**) Experimental design of the MI treatment model. (**b**) Doppler echocardiography in M-mode of the left ventricle at the fourth week posttreatment. (c–h) Echocardiographic evaluation of cardiac function in the sham group (*n* = 5), the MgIV group, the MgAP treatment group, and the untreated MI group (*n* = 7–9) at the first week and fourth week. The data are representative of independent experiments. All data are expressed as the means ± SDs; ns: nonsignificant, *P* > 0.05; ^*^P < 0.05; ^**^P < 0.01; ^***^P < 0.001; ^****^P < 0.0001. Statistical analysis for comparisons between two groups was performed using Student two-tailed t-test. For comparisons involving more than two groups, statistical significance was assessed using a one-way ANOVA followed by an LSD test

Within the field of MI treatment, notable progress has been made; yet restoring complete cardiac function and enhancing myocardial cell regeneration continue to be significant challenges. This study explored a therapeutic strategy aimed at increasing local Mg^2+^ concentrations at MI sites. We evaluated the therapeutic effectiveness of a cardiac patch (MgAP) designed for the gradual release of Mg^2+^ in a rat MI model. The MI model was established in SD rats by ligating the LAD artery. Cardiac function and remodeling post-MI were assessed using Doppler echocardiography and histological evaluations on Days 7 and 28 (Figure 3a). After 4 weeks of treatment, changes in cardiac functionality among the different treatment groups were assessed using left ventricular parasternal long-axis echocardiography in M-mode (Figure 3b). The LVEF and LVFS are commonly used to evaluate cardiac function, as they reflect the heart’s pumping efficiency and contractility [38]. Compared with the MI control group, the groups treated with intravenous Mg (MgIV) and MgAP demonstrated significant improvements in LVEF and LVFS. Specifically, the MgIV group showed improvements of 9.3 ± 3.6% in LVEF and 5.8 ± 2.2% in LVFS. The MgAP group showed even greater improvements, with the LVEF increasing by ~20.3 ± 6.5% and the LVFS increasing by 13.6 ± 5.1% (Figure 3c–f). Additionally, significant reductions in left ventricular internal diameters were observed, with the MgIV group showing a decrease of ~1.2 ± 0.5 mm in the systolic internal diameter, indicating markedly reduced cardiac dilation. This reduction was even more significant in the MgAP group, with a decrease of 2.1 ± 0.4 mm (Figure 3g and h). These results confirm the efficacy of MgIV and MgAP in improving cardiac function and reducing cardiac dilation, demonstrating the potential of these treatments to enhance cardiac remodeling post-MI.

#### The MgAP elastic patch elevated myocardial strain and synchrony post-MI injury

**Figure 4:**
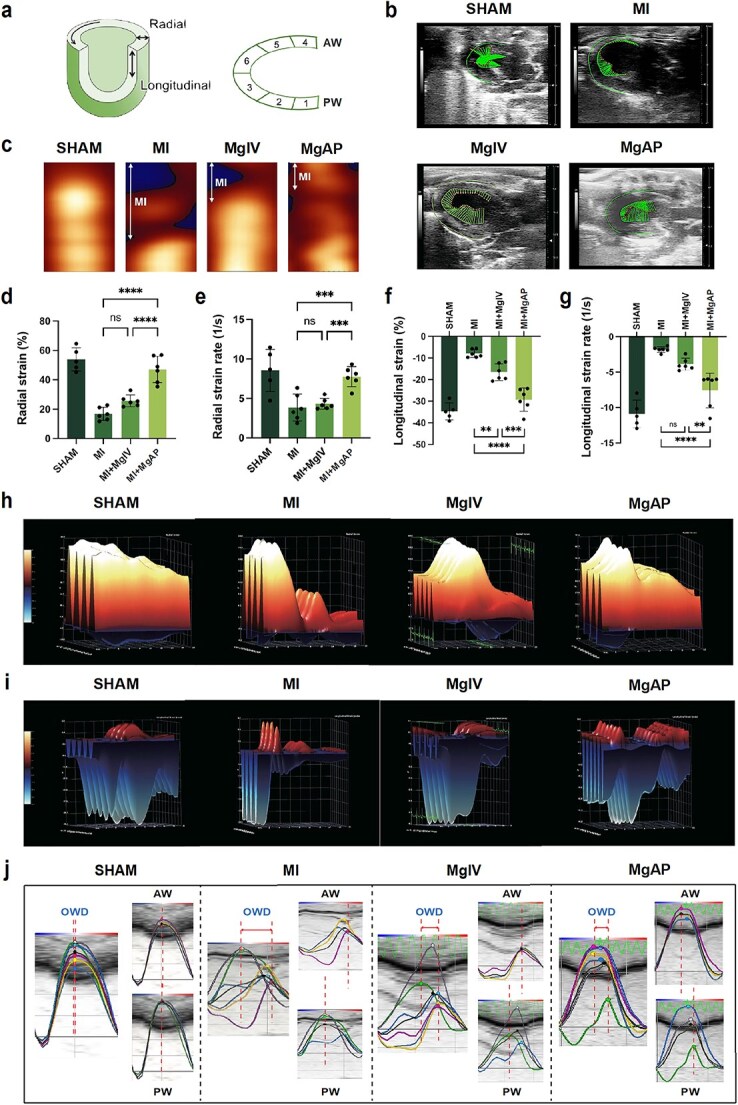
MgAP improved cardiac strain and synchrony in the rat MI model. (**a**) Left: schematic diagram of radial and longitudinal deformation of the left ventricle. Right: variation tendency of different myocardial segments shown by speckle-tracking echocardiography. (**b**) Left ventricular parasternal longitudinal speckle-tracking echocardiography at the fourth week posttreatment. The length of the indicator line represents the degree of displacement. (**c**) Heatmap of the radial displacement of the ventricular wall. (**d**–**g**) Radial and longitudinal strain and strain rates of the left ventricular wall at the fourth week in each group (*n* = 6). (**h**) Representative three-dimensional diagram of radial strain. (**i**) Representative three-dimensional diagram of longitudinal strain. (**j**) Representative schematic diagrams of OWD in the fourth week. (AW: The anterior wall area of left ventricle; PW: The posterior wall area of left ventricle). The data are representative of independent experiments. All data are expressed as the means ± SDs; ns: nonsignificant, *P* > 0.05; ^**^P < 0.01; ^***^P < 0.001; ^****^P < 0.0001. Statistical significance was calculated by one-way ANOVA with the LSD test

The assessment of LV systolic function is the primary method used to judge cardiac function. However, it cannot reflect the dynamics of cardiac deformation, which involves complex twisting and untwisting movements, longitudinal compression, circumferential narrowing, radial expansion, and diastolic elongation and relaxation [39, 40]. These movements are crucial for effectively ejecting blood during systole and refilling the heart chambers during diastole, highlighting their essential role in maintaining cardiac function. Strain and strain rate are recognized as important indicators for the early detection of myocardial abnormalities [41]. As depicted in Figure 4a, myocardial tissue deformation across the cardiac cycle is differentiated into longitudinal and radial dimensions. Longitudinal strain is considered a key prognostic indicator for predicting left ventricular contractile remodeling [42, 43]. Using speckle-tracking echocardiography, we divided the left ventricle into six regions, focusing on the fourth to sixth regions (the anterior wall area), which correspond to the areas of cardiac ischemic damage and intervention. Figure 4b displays the speckle-tracking diagrams for each group, with the length of the green tracking lines representing the degree of ventricular wall displacement. This capability of myocardial displacement is clearly illustrated in the displacement heatmaps (Figure 4c), which showed significantly greater myocardial wall displacement in the MgAP-treated group than in the groups with impaired movement, indicating a greater capacity for myocardial movement. Consistent with the echocardiographic findings, our statistical analysis of the changes in radial and longitudinal strain revealed that, compared with the MI control group, the MgAP-treated group exhibited significant improvements of ~27.4 ± 4.1% in radial strain and 20.7 ± 3.9% in longitudinal strain, outperforming the MgIV group. The MgAP treatment group also presented positive trends in the rates of radial and longitudinal myocardial deformation (Figure 4d–g). Through three-dimensional simulations, changes in myocardial strain, both radial and longitudinal, are clearly visible (Figure 4h and i), with animals treated with MgIV and MgAP showing substantial biomechanical strain improvements compared with those in the MI control group. These findings indicate that while both materials significantly enhanced the biomechanical properties of the cardiac wall, MgAP produced superior effects. To further explore changes in synchrony between the contractions of the anterior and posterior walls of the heart, we analyzed the variation in peak strain times of the left ventricular wall, specifically the opposing wall delay (OWD). In the sham group, the heart strain trajectories presented minor synchrony delays, demonstrating well-coordinated, normal cardiac contractions. In contrast, a significant increase in OWD post-MI indicated notable disruptions in contraction synchrony. Impressively, treatment with MgAP implants resulted in a significant reduction in OWD, leading to more consistent motion trajectories across cardiac segments, suggesting effective restoration of cardiac synchrony (Figure 4j).

### The MgAP elastic patch mitigated adverse myocardial remodeling in the rat MI model

**Figure 5:**
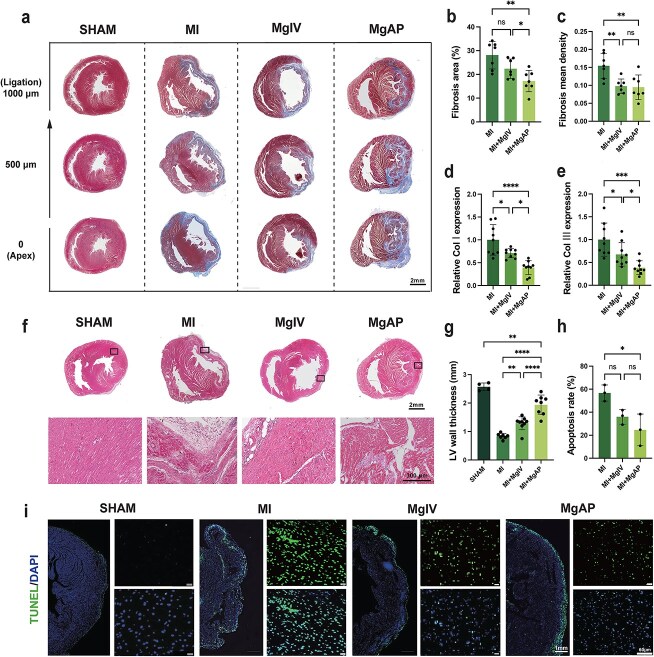
MgAP treatment mitigated adverse myocardial remodeling in the rat MI model. (**a**) Representative Masson trichrome staining of the heart at the fourth week; (**b**) analysis of the fibrotic area; and (**c**) mean fibrotic density. (**d** and **e**) qPCR was used to detect the mRNA expression of collagen I/collagen III. (**f**) Representative H&E staining of the heart at the fourth week and (**g**) analysis of left ventricular wall thickness. (h and i) DAPI/TUNEL staining of the MI area at the fourth week and quantitative analysis. The data are representative of independent experiments. All data are expressed as the means ± SDs; ns: nonsignificant, *P* > 0.05; ^*^P < 0.05; ^**^P < 0.01; ^***^P < 0.001; ^****^P < 0.0001. Statistical significance was calculated by one-way ANOVA with the LSD test

Following MI, the proliferation and activation of myofibroblasts and the extensive production of collagen lead to progressive replacement fibrosis, causing detrimental changes in cardiac structure and function [44]. To assess the extent of myocardial fibrosis 4 weeks after treatment, we utilized Masson trichrome staining. Our results showed that treatment with both MgIV and MgAP significantly reduced collagen accumulation. Notably, MgAP treatment decreased the fibrotic area by ~10.9 ± 1.2%, effectively reducing the size of the fibrotic zone within the infarcted region and thus diminishing pathological cardiac remodeling (Figure 5a–c). Further analysis of collagen I and collagen III mRNA levels in cardiac tissues revealed trends consistent with the Masson trichrome staining results (Figure 5d and e). Additionally, H&E staining for morphological examination revealed that the myocardial wall thickness in the infarcted region in the MgAP group remained stable at ~1.9 ± 0.3 mm (Figure 5f and g), underscoring the importance of MgAP in maintaining myocardial structural integrity crucial for normal heart function. Moreover, TUNEL staining revealed the significant effectiveness of MgAP in reducing myocardial cell apoptosis, with a decrease in cardiac cell apoptosis rates of 32.1 ± 5.5% (Figures 5h and i and S2), highlighting its protective effect on the myocardium.

### Transcriptomic exploration of the molecular mechanisms underlying the improvement in MI caused by the MgAP elastic patch

**Figure 6:**
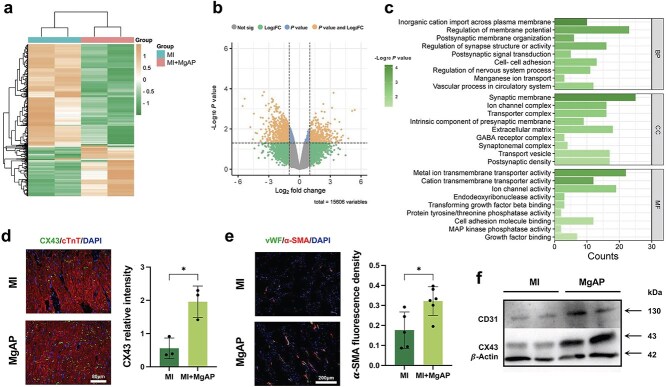
Transcriptomic analysis and validation of MgAP-mediated repair mechanisms. (**a**) Heatmap of differentially expressed genes between the MI + MgAP group and the MI group. (**b**) Volcano map of all the expressed genes in the MgAP group and MI group (*n* = 2). (**c**) Representative GO analysis of significant variation in biological process (BP), cell component (CC), and molecular function (MF) terms in the MgAP group. (**d**) Representative images of immunofluorescence staining and quantitative analysis of DAPI, CX43, and cTnT in cardiac sections from animals in the MI and MI + MgAP groups at the fourth week. (**e**) Representative images of immunofluorescence staining and quantitative analysis of DAPI, vWF, and α-SMA in cardiac sections from animals in the MI and MI + MgAP groups. (**f**) Western blot analysis of cardiac CX43 and CD31 protein levels in the MI group and MI + MgAP group after 4 weeks of treatment. (CX43: Connexin 43; CD31: Cluster of differentiation 31). All data are expressed as the means ± SDs; ns: nonsignificant, *P* > 0.05; ^*^P < 0.05. Statistical analysis for comparisons between two groups was performed using Student two-tailed *t*-test

Four weeks post-MI, we conducted transcriptomic analyses of heart tissues from the infarct zone using RNA sequencing to investigate gene expression changes in the pathological state of the heart. By employing heatmaps and volcano plots, we compared the mRNA expression levels between the MI and MI + MgAP groups, revealing differences influenced by the therapeutic interventions (Figure 6a and b). In the MgAP group, 557 genes were significantly upregulated, whereas 1209 genes were significantly downregulated (cutoff = 1.25, *P* < 0.05). Magnesium ions are known to increase cardiac electrical stability, likely through the inhibition of calcium channels, reducing intracellular calcium levels and thereby affecting myocardial contractility [45, 46]. GO analyses of samples from the MgAP group revealed enrichment of pathways related to transmembrane inorganic ion transport, ion channel activities, membrane potential regulation, postsynaptic signaling pathways, and cell adhesion, among others. These findings suggest improvements in cardiac electrophysiological properties and cellular interconnectivity (Figure 6c). Importantly, enhancement of vascular system and circulatory processes in the MgAP group indicate that the treatment not only aids cardiac recovery but also positively impacts the overall health of the circulatory system, enhancing the blood supply and promoting angiogenesis. These findings underscore the treatment’s multifaceted potential mechanisms for cardiac recovery and functional improvement.

The expression level of CX43, a critical gap junction protein, has been reported to be crucial for myocardial electrical signal transmission and enhancing cardiac conduction synchronization [47, 48]. As shown in Figure 6d, MgAP treatment increased the expression of CX43 at the infarct border, which could enhance the interaction between cardiomyocytes. Furthermore, immunofluorescence staining revealed increased expression of the vascular markers von Willebrand factor (vWF) and alpha-smooth muscle actin (α-SMA) in the peri-infarct area after MgAP treatment, indicating increased vascular density (Figure 6e). Western blot analysis further confirmed the significant upregulated protein expression of CX43 and the vascular marker CD31 following MgAP treatment, providing additional evidence for the potential repair mechanism of MgAP (Figure 6f).

### The MgAP elastic patch enhanced angiogenesis in the limb ischemia model

**Figure 7:**
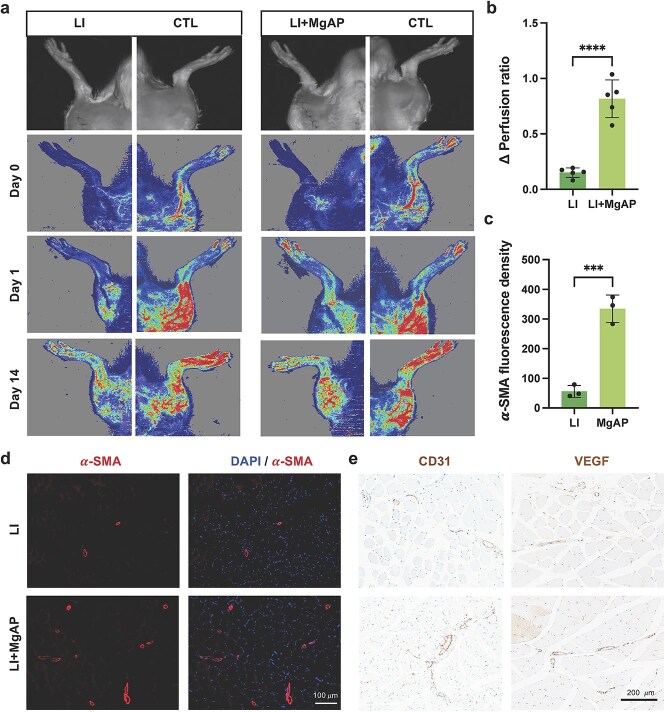
The MgAP patch enhanced angiogenesis to repair ischemic tissue. (**a**) A Doppler laser scanner was used to quantify blood flow by imaging both limbs in rats before (Day 0) and after treatment (Day 14). (**b**) Increase in the perfusion ratio between Day 14 and Day 0 (∆perfusion ratio). (**c** and **d**) Representative images of immunofluorescence staining and quantitative analysis of DAPI and α-SMA in the lower LI sites of animals in the LI and LI + MgAP groups. (**e**) Representative CD31 and VEGF immunohistochemical staining of limb muscles in the LI and LI + MgAP rat models. All data are expressed as the means ± SDs; ns: nonsignificant, *P* > 0.05; ^***^P < 0.001; ^****^P < 0.0001. Statistical analysis for comparisons between two groups was performed using Student two-tailed *t*-test

Treatment strategies for ischemic conditions, whether for the heart or peripheral tissues, typically focus on improving blood flow and tissue perfusion. Initially explored for myocardial ischemia, MgAP has also demonstrated promising results in treating lower LI. This study evaluated the ability of MgAP to promote angiogenesis in a rat model of LI established by ligating the femoral artery. Blood flow in both limbs was measured with a Doppler laser scanner before (Day 0) and after treatment (Day 14) to evaluate perfusion in the ischemic versus healthy limbs. Compared with the LI group, the LI + MgAP group presented significant improvement in blood perfusion in the ischemic limbs (Figure 7a and b). DAPI staining (nuclear marker) and immunofluorescence staining for α-SMA (a marker of activated smooth muscle cells and myofibroblasts involved in vascular remodeling) were performed at the lower LI site in both the LI and LI + MgAP groups (Figure 7c and d). The results revealed a significantly greater α-SMA-positive cell density in the MgAP-treated group than in the LI group, indicating enhanced vascular remodeling and smooth muscle cell activation. Furthermore, immunohistochemical staining for the vascular markers CD31 (an endothelial cell-specific marker) and VEGF (vascular endothelial growth factor, a key regulator of angiogenesis) in the LI model revealed markedly increased protein expression in the MgAP-treated group (Figure 7e). These findings collectively suggest that MgAP promotes robust angiogenic activity, leading to improved local circulation and effective repair of ischemic tissue.

## Discussion

In the context of ischemic injury, restoring organ function and limiting pathological remodeling remain major challenges. This study demonstrates the successful development of an MgAP, which facilitates the localized and sustained delivery of Mg^2+^ ions to the ischemic myocardium and limb tissue. We observed a remarkable therapeutic effect of the patch in terms of promoting revascularization in a related *in vivo* ischemia model. This novel MgAP patch offers significant advantages over traditional systemic magnesium administration routes, such as intravenous or oral routes, by ensuring targeted delivery, minimizing side effects, and enhancing therapeutic efficacy.

In the past several decades, diverse types of natural polymers, such as starch, cellulose, collagen, chitosan, sodium alginate, hyaluronic acid, and xanthan gum, have been utilized to prepare biological hydrogels [49]. Among these natural polymer-based hydrogels, starch-based hydrogels have emerged as promising biomaterials and have been applied for the repair and regeneration of multiple tissues, such as bone [50], cartilage [51], skin [52], muscle [53], and myocardium [54], owing to their advantages of low cost, high biosafety, high biodegradability, and easy tunability of physical properties. In addition, the porous molecular network of starch-based hydrogels creates a biomimetic extracellular matrix-like structure, providing a supportive environment for the adhesion, proliferation, and differentiation of cells [55]. However, the current problems associated with starch-based hydrogels for tissue regeneration are insufficient bioactivity and rapid degradation properties due to the hydrolysis of amylase *in vivo*. To address these problems, our work optimized the bioactivity and degradation properties of starch-based hydrogels by introducing bioactive Mg^2+^ ions and constructing the ionic complex MgAP. MgAP not only has a degradation rate suitable for supporting tissue regeneration but also exhibits stronger adaptive adhesion to ischemic sites with varying geometries, allowing it to reliably exert biological effects.

Magnesium is an essential element in numerous physiological processes, including the regulation of vascular tone, endothelial cell function, and ion channel activity [56, 57]. Magnesium ions have been shown to enhance endothelial cell proliferation, migration, and tube formation, which are critical steps in the formation of new blood vessels [58–60]. Our results confirmed these findings, as MgAP significantly promoted proliferation and tube formation by HUVECs *in vitro* and effectively enhanced angiogenesis and blood vessel formation in ischemic tissues *in vivo*. Additionally, the results from our *in vivo* experiments in MI and lower LI models demonstrated the efficacy of MgAP in enhancing revascularization. As shown in Figure S3a and b, we noticed that magnesium ion levels decreased immediately following infarct injury. The administration of the MgAP patch effectively elevated the Mg^2+^ level in the myocardium. The local Mg^2+^ level was maintained at the same level from Day 1 to Day 5. This result indicated that magnesium ions were released from the MgAP patch in a sustained manner. Moreover, the Mg^2+^ level in the serum did not show significant changes on either Day 1 or Day 5 (Figure S3c and d), which verified that our local delivery strategy was effective and could avoid the drawbacks of traditional intravenous or oral magnesium administration. Notably, the local Mg^2+^ level in the lower limb muscle did not change as much as that in the myocardium did (Figure S3e and f). We considered that the constant contraction and relaxation of myocardial tissue could have accelerated the release of magnesium ions from the MgAP patch. Transcriptomic analysis of cardiac tissues was subsequently performed. The upregulation of connexin 43 (CX43), a key protein involved in cardiac conduction and gap junction communication, suggests that MgAP may help restore myocardial electrical stability and improve synchronization among cardiomyocytes [61]. The increased expression of vascular markers, including vWF and α-SMA, in the peri-infarct region after MgAP treatment further supports its role in promoting angiogenesis and improving vascular density. Given the positive outcomes of MgAP in the myocardial and LI models, exploring its utility in the treatment of other tissue types could broaden its potential therapeutic applications. As reported, Mg^2+^ can facilitate osteogenic differentiation and modulate neuronal ion channels [62, 63]. This evidence from cross-disciplinary applications of Mg^2+^-based biomaterials could expand the potential applications of MgAP to include bone regeneration or neural repair therapies.

Great progress has been made in conventional therapies for ischemic diseases, such as pharmacological treatment and interventional or revascularization surgery, and emerging strategies under development include gene therapy, recombinant protein therapy, or stem cell-based approaches. Gene therapy and recombinant proteins offer precise therapeutic targets but face issues such as high cost, gene delivery challenges, and immune rejection risks [64–66]. Stem cell-based therapies hold potential for tissue regeneration but are hindered by poor cell survival rates, potential tumorigenicity, and complex clinical logistics [67–69]. In comparison, MgAP offers a more accessible, cost-effective solution. Unlike gene and protein therapies, MgAP does not rely on biological agents, reducing the risk of immunogenic reactions. Additionally, its ease of implantation and ability to provide localized treatment without complex biological processes give it a unique advantage in clinical settings. The simplicity of MgAP could make it a viable treatment for a broad range of ischemic diseases, without the extensive costs and technical barriers posed by other advanced therapies. Moreover, tissue engineering has gained increasing attention as a field that can address the multifaceted challenges of ischemic diseases [70–73]. Our findings align with those of similar studies that have tested bioactive ion-releasing scaffolds or growth factor-based treatments for ischemia [74, 75]. However, the bioadaptive properties of MgAP also provide additional therapeutic benefits. Its viscoelastic nature allows it to adapt to tissue deformation, reducing mechanical stress and preventing pathological remodeling, a common complication observed with nonbioadaptive materials [76, 77]. Compared with conventional methods, this combined approach of bioactive ion release and mechanical support offers a more effective strategy for tissue repair.

In terms of clinical translation, the next step for the use of MgAP involves preclinical studies in large animals to evaluate its safety, biocompatibility, and optimal dosage. The clinical implementation of MgAP will also require optimization of implantation techniques to accommodate different anatomical sites and minimize individual variability in treatment outcomes. Challenges such as tissue response, long-term stability, and potential allergic reactions must be addressed to ensure successful clinical use. Nonetheless, the encouraging results from our study suggest that with proper refinement, MgAP could significantly improve the treatment of ischemic diseases.

## Conclusions

In conclusion, MgAP represents a promising and innovative approach for the localized delivery of magnesium ions to ischemic tissues. By enhancing angiogenesis, improving myocardial function, reducing fibrosis, and protecting against myocardial apoptosis, MgAP provides a multifaceted strategy for the treatment of ischemic diseases. Our results highlight the significant potential of MgAP in both MI and lower LI, suggesting that this therapy could be applied to a wide range of ischemic conditions. Further clinical studies are necessary to explore the long-term safety and efficacy of MgAP, but this novel biomaterial holds great promise for advancing the treatment of ischemic diseases.

## Supplementary Material

Supplementary_Materials-A_bioactive_Hydrogel_Tissue_Repair_tkaf005
