# Supplementary material for: A bioactive hydrogel patch accelerates revascularization in ischemic lesions for tissue repair
Source: Burns Trauma. 2025 May 2;13:tkaf005. doi: 10.1093/burnst/tkaf005 (PMC12048007; doi:10.1093/burnst/tkaf005)
Supplement: Supplementary_Materials-A_bioactive_Hydrogel_Tissue_Repair_tkaf005 [file supplementary_materials-a_bioactive_hydrogel_tissue_repair_tkaf005.docx]

Supplementary Materials for

**A bioactive Hydrogel Patch Accelerates Revascularization in Ischemic Lesions for Tissue Repair**

Zhuo Liu^1,6^**^†^**, Kang Wu^1^**^†^**, Hong Zeng^1^**^†^**, Wenxin Huang^1^, Xuemeng Wang^1^, Ying Qu^1^, Chuntao Chen^3^, Lei Zhang^3^, Dongpin Sun^3^, Sifeng Chen^1^, Xiao Lin^2^*, Ning Sun^1,4^*, Lei Yang^2,5^*, Chen Xu^1^*.

* Corresponding authors: xuchenfd@fudan.edu.cn (C. Xu); [ylei@hebut.edu.cn](mailto:ylei@hebut.edu.cn) (L. Yang); [sunning@jiangnan.edu.cn (N](mailto:sunning@jiangnan.edu.cn%20(N). Sun); [xlin@suda.edu.cn (X](mailto:xlin@suda.edu.cn%20(X). Lin)

**The PDF file includes:**

Figure. S1 to S3


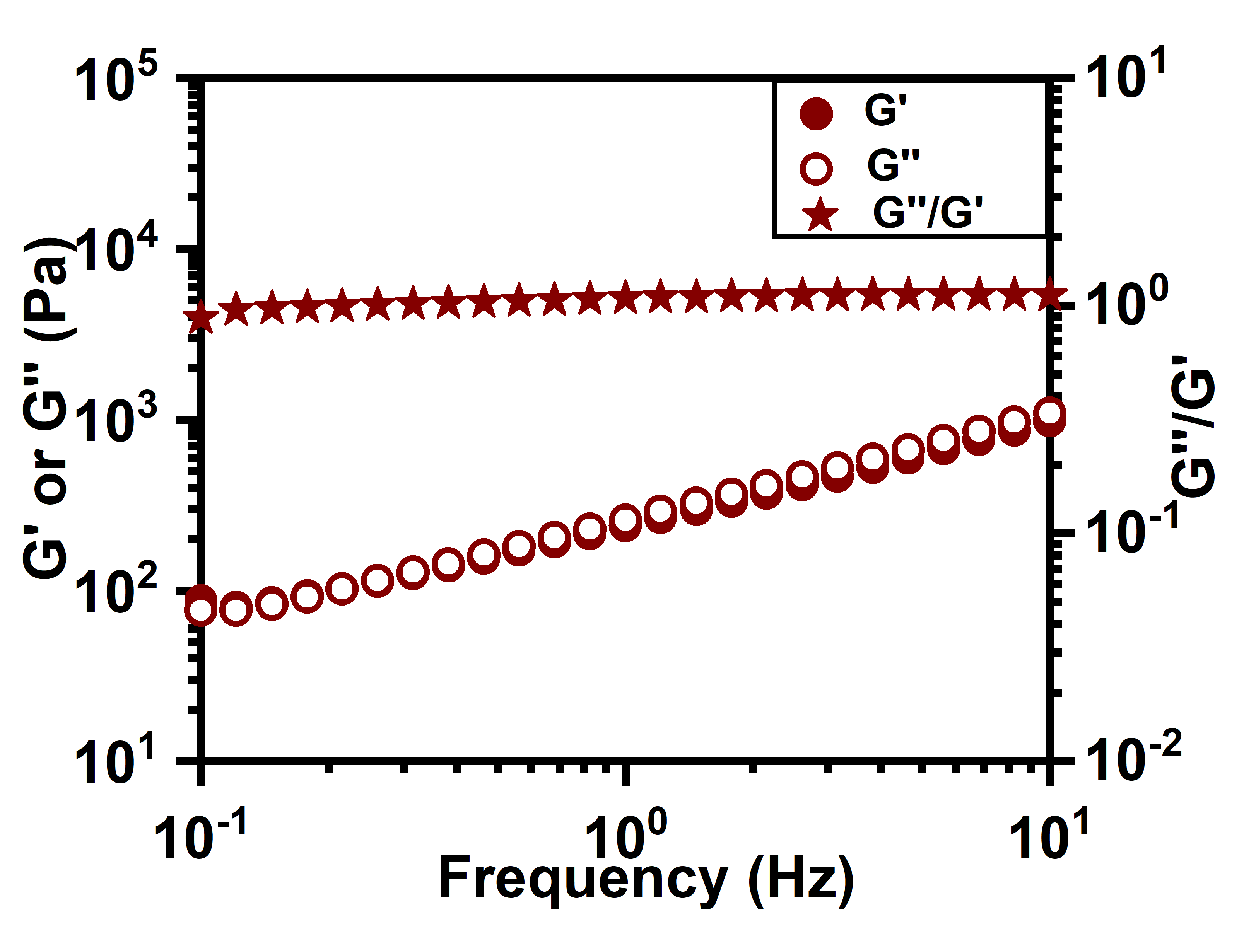


**Figure S1.** **Dependence of Gʹ, Gʹʹ, and the Gʹʹ/ Gʹ ratio of MgAP on the frequency of oscillation after** **sealed storage for 60 days.**


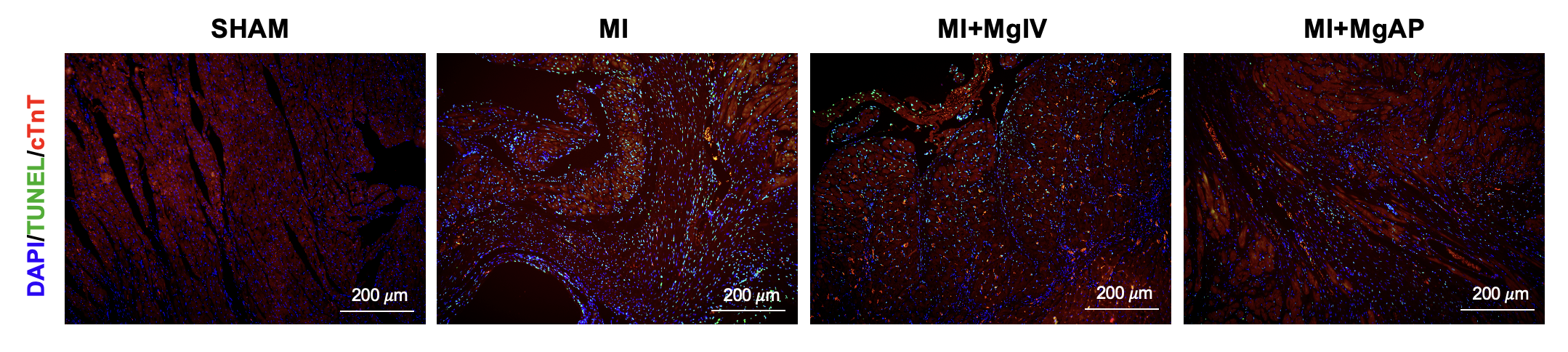


**Figure S2.** **Representative staining of DAPI (blue) /TUNEL (green) /cTnT (red) in cardiac sections of SHAM, MI, MI+MgIV and MI+MgAP group at 4^th^-week.**

**Figure S3. The concentration of Mg^2+^ in the extracted solution of heart tissue and limb tissue, and blood samples in MI/LI rat models for 1 day or 5 days.** (a-b) The mass ratio of Mg^2+^ in heart tissue of MI rat models on day 1 and day 5, respectively. (c-d) The concentration of Mg^2+^ in blood serum of MI rat models on day 1 and day 5. (a-b) The mass ratio of Mg^2+^ in limb tissue of LI rat models on day 1 and day 5. Data are representative of independent experiments. All data are expressed as mean ± SD; *ns*: non-significant, *P* > 0.05; **P* < 0.05; ***P* < 0.01; ****P* < 0.001; *****P* < 0.0001; Statistical significance was assessed using a one-way ANOVA followed by an LSD test.
